# A response to yet another defence of ECT in the absence of robust efficacy and safety evidence

**DOI:** 10.1017/S2045796021000846

**Published:** 2022-02-15

**Authors:** John Read

**Affiliations:** School of Psychology, University of East London, Water Lane, Stratford, London E15 4LZ, UK

**Keywords:** adverse effects, efficacy, electroconvulsive therapy, ethics, placebo, systematic reviews, depression

## Abstract

It is estimated that electroconvulsive therapy is still administered to approximately a million people a year. It involves passing enough electric current through the human brain, eight to twelve times, to cause convulsions, in the hope of somehow alleviating emotional suffering, primarily depression. There have only ever been 11 placebo-controlled studies (where general anaesthesia is administered but the electric shock is withheld), all of which were pre-1986, had very small sample sizes and were seriously methodologically flawed. Five of these studies found no difference between the two groups at the end of treatment, four found ECT produced better outcomes for some patients, and two produced mixed results, including one where psychiatrists' ratings produced a difference, but the ratings of nurses and patients did not. In the 80 years since the first ECT no studies have found any evidence that ECT is better than placebo beyond the end of treatment. Nevertheless, all five meta-analyses relying on these studies have somehow concluded that ECT is more effective than placebo despite the studies' multiple failings. Meanwhile, evidence of persistent or permanent memory loss in 12% to 55% of patients has accumulated. Attempts to highlight this failure of ECT proponents to provide robust evidence that their treatment is effective and safe are routinely dismissed, diminished, denied and denounced. This paper responds to one such attempt, by Drs Meechan, Laws, Young, McLoughlin and Jauhar, to discredit two systematic reviews of the eleven pre-1986 studies, in 2010 and 2019, the latter of which also reviewed five meta-analyses that had ignored the studies' failings. The criticisms and claims of the recent crtiique of the two systematic reviews are examined in detail, by the first author of both reviews, for accuracy, relevance and logic. The critique is found to include multiple errors, misrepresentations, omissions, inconsistencies and logical flaws. It is concluded that Meechan *et al*. fail to make a fact-based, coherent argument against suspending ECT pending a series of large, carefully designed placebo-controlled studies to establish whether ECT does have any beneficial effects against which to weigh the significant established adverse effects.

## Two reviews of ECT for depression

I thank Chis Meechan *et al*. for focusing attention on two systematic reviews of randomised placebo-controlled trials (RCTs) of the efficacy of ECT for depression (Read and Bentall, 2010; Read *et al*., 2019*a*), the latter of which also reviewed the five meta-analyses of those studies. Both reviews had concluded that the RCTs, all small pre-1986 studies, were so flawed that no conclusions about efficacy can be based on them, and that the meta-analyses failed to take into account these flaws, and ignored the absence of any RCT evidence of positive outcomes beyond the end of treatment, when claiming that ECT is effective.

Before documenting some of the specific errors, misrepresentations, biases and omissions in Meechan *et al*.'s critique of the two reviews, we note that every single flaw in the two reviews alleged in their paper supposedly minimised the efficacy, or exaggerated the risks, of ECT. Conversely, they do not raise a single error, in either review, that might have exaggerated the efficacy or minimised the risks. Indeed, Meechan *et al*. fail to identify a single redeeming feature anywhere in the 15 pages of the 2010 review or the 41 pages of the 2019 review. Their critique is unremittingly one-sided, in favour of defending ECT.

## Meechan *et al*.'s general strategies to discredit the reviews

Most of the criticisms Meechan *et al*., make of our 2019 review have been repeatedly raised before, often in quite vitriolic terms, and have been repeatedly rebutted: Andrade (2021) – refuted by Read (2021*a*), Anderson (2021) – corrected by Read (2021*b*), Gergel *et al*. (2021) – rejected by Read *et al*. (2022), Henry (2021) – countered by Hancock *et al*. (2021); and Kirov *et al*. (2021) – negated by Read *et al*. (2021*a*). Some of the criticisms were also addressed in a podcast involving Professor Irving Kirsch, of Harvard Medical School, co-author of the 2019 review (Moore, 2020). None of these previous corrections of, or rebuttals to, the points they repeat in their own attack on our work are mentioned by Meechan *et al*. in their paper.

Meechan *et al*. even cite two of these five previous critiques (Andrade, 2021; Kirov *et al*., 2021) as corroborating evidence for their own opinions (without mentioning the rebuttals). Furthermore, two of Meechan's co-authors (Drs Declan McLoughlin and Sameer Jauhar) contributed to the piece in the *British Journal of Psychiatry*, entitled ‘ECT for depression: 80 years of progress’ (Kirov *et al*., 2021). It made some of the same claims recycled in the Meechan *et al*. paper. The brief rebuttal, by seven psychiatrists and three research professors, had space to document only ‘a few of the errors, omissions, and distortions’ (Read *et al*., 2021*a*). Fortunately, I have more space here.

### Narrative or systematic?

Meechan *et al*.'s central strategy for dismissing the two reviews, and their inconvenient findings, is to portray them as narrative, rather than systematic, reviews. This point is so central to their mission that the term is highlighted in their title, *‘A critique of narrative reviews of the evidence-base for ECT in depression’,* repeated in all four subsections of their Abstract, and then used seven times in the body of their article, including, at the very end: ‘we have identified numerous substantial problems that stem from these narrative reviews’. Despite this repetitive insistence that both reviews are narrative rather than systematic, Meechan *et al*. go on to conduct elaborate evaluations of them using AMSTAR2, which is ‘a critical appraisal tool for *systematic* reviews’ (Shea *et al*., 2017). Readers would be unaware of this contradiction, however, as they were told that AMSTAR2 is a tool ‘to assess quality of reviews’ in general.

Given the miniscule size of the body of literature in question, the issue of narrative vs. systematic is, any way, irrelevant. Nobody, including Meechan *et al*., disputes the fact that there have only ever been ten or 11 randomised placebo-controlled trials (RCTs) of ECT for depression (depending on whether Ulett *et al*. (1956) is counted; see Read and Bentall, 2010, p. 336; Read *et al*., 2019*a*, pp. 69, 70)). We all agree there have been none since 1985.

Furthermore, both narrative and systematic reviews can be valuable in different contexts (Collins and Fauser, 2005). Although there may not be complete consensus about their definitions and the differences between them (Collins and Fauser, 2005; Pae, 2015), the absolutely essential characteristics of systematic reviews are that they must do everything reasonably possible to include *all* relevant publications, must transparently define what the reviewers consider relevant (i.e. state inclusion criteria) and must clearly describe an effective search strategy. As we shall see, both reviews in question (Read and Bentall, 2010; Read *et al*., 2019*a*) met all of these criteria, thereby meeting the vital requirement, for systematic reviews, of replicability.

While attempting to relegate the two reviews whose findings challenge the efficacy of ECT to an inferior ‘narrative’ status, reviews which claim that ECT is effective are positioned by Meechan *et al.*, as superior, because they are, supposedly, ‘conventional’, ‘systematic’ and ‘standardised’. Ironically, Meechan *et al*. could have set out to conduct a systematic review critiquing *all* reviews of the literature, but chose instead to conduct a highly selective, narrative, review of just two, the two whose findings they don't like.

One of the two primary goals of the Read *et al*. (2019*a*) review was to assess the quality of the meta-analyses that had analysed the 11 RCTs. All the meta-analyses had been shown to be flawed and biased. By attempting to position these meta-analyses as superior just by labelling them ‘systematic’ ‘conventional’ and ‘standardised’, without critiquing them in any way, while simultaneously attempting to dismiss a systematic review that did evaluate the meta-analyses (comprehensively, including a detailed Table), by wrongly labelling it ‘narrative’, seems, at best, disingenuous.

Given that the senior author (Professor Allan Young) on the most recent of these meta-analyses (Mutz *et al*., 2019) is a co-author of the Meechan *et al*. paper that makes such strong allegations about our being ‘selective’, it is worth noting that Young's supposedly systematic meta-analysis was *so* selective it selected only one of the 11 studies (Brandon *et al*., 1984) when calculating efficacy. Reasons for not selecting the other ten varied, but included ‘Cannot be obtained’, a category which they applied to two studies published in the Lancet (Freeman *et al*., 1978; Johnstone *et al*., 1980). When asked ‘Does the Institute of Psychiatry not have access to the Lancet’, no answer was forthcoming (Read *et al*., 2019*a*, p. 89). In fact, Young's meta-analysis (Mutz *et al*., 2019) claimed to have determined that two types of ECT were superior to sham ECT (and that two types were not), even though the only study they selected had only involved one of those four types (Read *et al*., 2019*a*, p. 89). Furthermore, this meta-analysis (like most of the other meta-analyses) selectively ignored all studies assessing whether ECT has any long-term benefits beyond the end of treatment. Perhaps this is because no studies have ever produced any evidence of such benefits. It seems Meechan *et al*., including Professor Young, have been both selective and inaccurate when it comes to deciding which reviews are selective and which are not.

Meechan *et al*. are also rather loose with their use of the generic term ‘review’. They describe two of the previously discussed earlier versions of their own paper (Andrade, 2021; Kirov *et al*., 2021) as ‘recently published reviews'. They were not reviews at all. They were extremely selective and targeted commentaries.

### Evaluated and approved?

Meechan *et al*.'s secondary opening gambit is:

ECT has been evaluated and approved by the National Institute for Health and Care Excellence (NICE) in the UK (NICE, 2003) and Food and Drug Administration (FDA) in the USA (FDA, 2018) and various international professional organisations (Bennabi *et al*., 2019; Malhi *et al*., 2020).

This conveniently superficial version of reality might lead an uninformed reader to assume that NICE and the FDA have issued some sort of general endorsement of ECT. In reality, both authorities recommend that ECT only be used in quite rare and highly specified circumstances, relating to, amongst other things, the severity of depression, imminent risk of suicide and failure of previous treatment approaches. Both are clear that it has no long-term benefits. NICE (2003) states:

It is recommended that electroconvulsive therapy (ECT) is used only to achieve rapid and short-term improvement of severe symptoms after an adequate trial of other treatment options has proven ineffective and/or when the condition is considered to be potentially life-threatening, in individuals with:

- severe depressive illness
- catatonia
- a prolonged or severe manic episode.

This very limited, circumspect approval is qualified even further, by the major risks of cognitive impairment, which are also not mentioned by Meechan *et al*.:

The decision as to whether ECT is clinically indicated should be based on a documented assessment of the risks and potential benefits to the individual, including: the risks associated with the anaesthetic; current co-morbidities; anticipated adverse events, particularly cognitive impairment; and the risks of not having treatment. (NICE, 2010).

Given Meechan *et al*.'s attempt to cite NICE in support of their critique of the two reviews, it seems important to point out that NICE has, for nearly 20 years, been calling for exactly the same thing as the two reviews, i.e. better research that can actually determine whether ECT does work and precisely how unsafe it is:

Further research is urgently required to examine the long-term efficacy and safety of ECT, including its use as a maintenance therapy and its use in particular subgroups who may be at increased risk, for example older people, children and young people, and during pregnancy. … In addition to the use of appropriately validated psychometric scales, outcome measures should include user perspectives on the impact of ECT, the incidence and impact of important side effects such as cognitive functioning, and mortality. (NICE, 2003)

Similarly, Meechan *et al*.'s claim about FDA endorsement fails to inform readers that the FDA's (2020) regulation code 21(G) states that a notice must be displayed next to ECT machines warning that ‘The long term safety and effectiveness of ECT has not been demonstrated.’

It is equally revealing to read the only two references offered in support of the claim that ECT has been ‘evaluated and approved’ by ‘various international professional organisations’. One is a summary of official guidelines in Australia, which include most of the caveats, conditions and reservations of NICE and the FDA (Malhi *et al*., 2020). The other is a summary of guidelines by the French Association for Biological Psychiatry and Neuropsychopharmacology. It barely mentions ECT at all. It is described, very briefly, as a ‘fourth line strategy’ which is ‘never recommended as a first-line treatment for the initial major depressive episode, irrespective of the clinical severity or clinical features’ (Bennabi *et al*., 2019).

## ‘The effectiveness of electroconvulsive therapy: a literature review' – 2010

This peer-reviewed, systematic review, by the highly esteemed British Clinical Psychologist Professor Richard Bentall and myself, was published in this journal, 12 years ago (Read and Bentall, (2010). It has been cited 197 times (*Google Scholar, 21.12.2021*). To my knowledge no criticism of its methodology has ever been published, until Meechan *et al*. This, of course, does not mean our review is without limitations.

### Narrative or systematic?

Meechan *et al*. repeatedly refer to it as a ‘narrative review’. They attempt to justify this misrepresentation with another. They claim that ‘No clear study inclusion criteria are provided in Read and Bentall's narrative review.’ In reality, our review clearly stated:

*PsycINFO, Medline,* previous reviews and meta-analyses were searched in an attempt to identify all studies comparing ECT with simulated-ECT [SECT]’ (p. 333).

The search terms used to identify studies meeting this very clear inclusion criterion were also explicitly listed (p. 334). Meechan *et al*. do not identify any ECT-SECT studies missed by this clearly described search strategy. There are none.

### Effectiveness

Meechan *et al*. assert that the review was guilty of ‘cherry-picking of outcome measures’. This is ironic given that five of the 11 studies that ECT proponents like Meechan *et al*. use as evidence for ECT's effectiveness, had, themselves, only partially reported their outcome findings (Read *et al*., 2019*a*). Meechan *et al*. provide just one example to support their ‘cherry-picking’ allegation:

For example, the Northwick Park trial's primary outcome measure was the Hamilton Depression Rating Scale (HDRS); the study was powered based on this measure, which showed a statistically significant better outcome with real ECT (Johnstone *et al*., 1980). Rather than focusing on the primary outcome and this significant difference between groups, the authors highlight other secondary outcomes that failed to show statistical significance.

In fact, the Read and Bentall review accurately reported *both* the positive and negative findings of this important study (p. 335). The improvement seen by psychiatrists was not seen by the nurses or by the patients. The researchers themselves had not used the term ‘primary’ to describe any of the three groups of raters. I disagree with Meechan *et al*. that psychiatrists' ratings are necessarily more important, or ‘primary’, than those of patients or nurses.

This is how the researchers themselves had reported their results:

Patients in both groups improved considerably during the course of the treatment but the improvement was greater in the real-ECT group. The advantage of real over simulated ECT was not retained and at the one-month and six-month follow-ups the Hamilton scores of the two groups were almost the same. The Leeds self ratings [by patients] showed similar trends but these were never significant, and this was also true of the ratings by nurses. … The most striking finding is that the differences which were present at the end of the course of eight treatments had disappeared one month later and were undetectable also at six months.’ (Johnstone *et al*., 1980, pp. 1318, 1319).

The Read and Bentall review is criticised for ‘Lack of an effect size measure, i.e. meta-analysis’. The review did not claim to be a meta-analysis. It merely reported the RCTs and their findings. Meechan *et al*. are correct, however, to point out that we did not report sample sizes.

Meechan *et al*. decided to conduct their own meta-analysis. In doing so they ignored a central point of both reviews, and *the* central point of the latter (Read *et al*., 2019*a*), namely that the quality of the studies in question are so flawed that they do not warrant being meta-analysed. Our review had concluded, after a very thorough analysis of each study, and of all five meta-analyses of those studies, that:

Contrary to the claims by the authors of all five meta-analyses, the small number of studies, the small sample sizes and the plethora of fundamental methodological flaws of most of the studies, render it impossible to determine whether or not ECT is superior to SECT (Read *et al*., 2019*a*, p. 92).

Conducting yet another meta-analysis, on exactly the same studies, is akin to your pointing out to yout car mechanic that the tests they just used in assessing your car to be roadworthy were all 40 years out of date and hopelessly flawed, only for the mechanic to respond, ‘No problem, we'll have another look at those test results then’.

Furthermore, we have already noted that a co-author of the Meechan *et al*. paper, Allan Young, agrees about the validity of the studies. Young was the senior author on the most recent meta-analysis (Mutz *et al*., 2019) that had judged that only one of the studies (Brandon *et al*., 1984) was robust enough for inclusion. He and his colleagues had even assessed that study as having a ‘high risk’ of bias.

Besides relying on woefully flawed, pre-1986 studies, another similarity of the Meechan *et al*. meta-analysis with all the previous ones, is that it completely ignores Read and Bentall's findings that none of the RCTs had found significant differences beyond the end of treatment and that ‘There are no placebo-controlled studies evaluating the hypothesis that ECT prevents suicide, and no robust evidence from other kinds of studies to support the hypothesis’ (p. 333).

### Risks

In relation to memory loss, Meechan *et al*. claim that Read and Bentall only ‘cite a single systematic review of patients’ perspectives on ECT (Rose *et al*., 2003) that included seven studies measuring subjective memory impairment as evidence of memory dysfunction'. (Note that patients' reports of memory impairment are automatically positioned as ‘subjective’, presumably in contrast to psychiatrists' supposedly ‘objective’ ratings). They go on to claim that three of the seven studies reported by Rose *et al*. did not meet Rose *et al*.'s own inclusion criterion of being at least 6 months post-ECT. Nevertheless, the four that ‘asked specifically whether they had experienced persistent or permanent memory’ produced findings ranging from 29% to 55% (Rose *et al*., 2003, Table 3). The 55% finding came from a study conducted 3 years after treatment (Squire and Slater, 1983). The 29% finding was after 4–9 years (Freeman and Kendall, 1980). In the most recent, and largest study (*n* = 418) most participants (70%) had had ECT more than 6 years ago (79% more than 2 years ago); and 40% reported ‘permanent loss of past memories’ (Pedler, 2000). After 3, 4, 6 or 9 years the brain damage involved can reasonably be called permanent. None of these findings is mentioned by Meechan *et al*.

The brief section on memory dysfunction in the Read and Bentall paper did not claim to be based on a systematic search of the relevant literature (unlike the main section, on effectiveness). The review highlighted the Rose *et al*. paper precisely because it reported on studies of patients' reports rather than only reports by psychiatrists like most studies. This must be acknowledged as a form of bias on our part. We did, however, also cite five additional, more traditional, studies on retrograde amnesia, and six on anterograde amnesia, all of which found memory loss, mostly months or years after ECT. Perhaps the best designed of these studies was conducted by Professor Sackeim *et al*. (2007). As we reported (Read and Bentall, 2010, p. 343):

Despite repeated claims, for 50 years, that ECT is safe, the first large-scale prospective study of cognitive outcomes following ECT did not occur until 2007. Prominent ECT advocate Sackeim *et al*. (2007) found that autobiographical memory was significantly (*p* < 0.0001) worse than pre-ECT levels both shortly after ECT and six months later. At both times the degree of impairment was significantly related to the number of shocks. Women and older people (both of whom are given ECT more frequently) were particularly impaired. The impairment was also greater among those who received bi-lateral ECT rather than unilateral ECT (bilateral remains the most common form of ECT despite multiple previous findings of greater damage). Even using a conservative definition of two standard deviations worse than pre-ECT scores, 38 (12.4%) met the criterion for ‘marked and persistent retrograde amnesia’.

Meechan *et al*. make no mention of Sackeim's study. Nor do they mention the following quote, in the 2019 review, from my earlier review, with Norwegian researcher Roar Fosse, of EEG, PET, SPECT and fMRI studies.

We suggest that the temporarily improved scores on depression instruments following ECT reflect the combination of frontal and temporal lobe functional impairments and activation of the HPA axis and the mesocorticolimbic dopamine system. These effects as well as other detailed changes observed in structures such as the hippocampus appear consistent with those typically seen after severe stress-exposure and/or brain trauma. (Fosse and Read, 2013, p. 6)

Meechan *et al*. do mention, at great length, a systematic review published 9 years after the Read and Bentall review. This review (Jones and McCollum, 2019) ignored all four of the studies cited by Rose *et al*. (2003), finding ‘persistent or permanent memory loss’ in 29%, 30%, 40% and 55% of ECT recipients. It failed to mention the Rose *et al*. review at all.

Meechan *et al*. allege that the Read and Bentall review failed to report ‘seven subjective memory studies identified by Jones and McCollum and conducted prior to Read and Bentall's (2010) review’, adding that ‘four found improvement in subjective memory scores’. Only three of the seven, however, had followed up longer than a few weeks. Of the three that followed up for 6 months, two had found no difference (Frith *et al*., 1983; Berman *et al*., 2008). The third (Ikeji *et al*., 1999) had found ‘subjective memory complaints’ in 37% of the ECT group. Thus, if Read and Bentall had been conducting a systematic review of persistent/permanent memory loss (which we were not), they would have found two studies that did not fall within the range of the four studies reported by Rose *et al*. (29%–55%), and one that did (37%). The bias of Meacham *et al*., is further revealed by their not only failing to inform readers that the four pre-2010 studies claiming to show enhanced memory performance following ECT had followed up only for very short periods, but also by their failing to state that in one case (Schulze-Rauschenbach *et al*., 2005) the improvement, recorded after just 1 week, was ‘not statistically or clinically significant’ (Jones and McCollum, 2019).

Meacham *et al*. further criticise Read and Bentall for not including, in their brief discussion of long term damage to memory (pp. 342, 343), most of the studies included in a simultaneous review by Semkovska and McLoughlin (2010), which had somehow concluded, astonishingly, that ‘Cognitive abnormalities associated with ECT are mainly limited to the first 3 days posttreatment.’ Meechan *et al*. fail to inform readers that this review had excluded all studies of retrograde amnesia, the most common form of memory loss caused by ECT. Semkovska and McLoughlin (2010) dismissed, for instance, the highly regarded Sackeim study that had found ‘marked and persistent retrograde amnesia’ in one in eight patients after 6 months, on the curious basis of ‘data unavailability’ (p. 569).

The bias of Semkovska and McLoughlin is plain from the first page of their review. The very first sentence, as is so often the case with ECT research papers, asserts that ‘ECT is the most acutely effective treatment for depression’. They cite just one reference in support of their assertion, a 2003 review, which actually drew no such conclusion (UK ECT Review Group, 2003; and see Read *et al*., 2019*a*, pp. 87, 88). These unsubstantiated statements proclaiming the efficacy and safety of ECT are not limited to research papers. A recent audit of ECT patient information leaflets currently in use at ECT clinics, and by the Royal College of Psychiatrists, in England found multiple inaccuracies, including many leaflets that exaggerated efficacy and minimised risks (Harrop *et al*., 2021; Read, 2021*c*).

Semkovska and McLoughlin also claimed that ‘descriptive reviews agree that after 6 months no deficits persist’, as if the Rose *et al*. (2003) review and the studies reported therein, simply did not exist. I have already mentioned the 6-month Sackeim *et al*. (2007) findings. A recent study, in Sweden, reported, among the 57 patients who had improved during ECT, six cases of ‘prolonged amnesia’ lasting 3–6 months (11%), and three (5%) still ongoing at the final, 12 month, follow up point (Ekstrand *et al*., 2021). (The numbers for the 34 who did not improve were not reported).

One of the two authors of the heavily biased Semkovska and McLoughlin review, is a co-author of the Meechan *et al*. paper. Readers of their paper might not realise that MECTA, from whom Dr McLoughlin admits receiving money (Meechan *et al*., 2021), is one of two US corporations that make ECT machines. MECTA filed for bankruptcy in 2021 because so many lawsuits had been filed against it that it could no longer obtain insurance cover.

## ‘Electroconvulsive therapy for depression: a review of the quality of ECT versus sham ECT trials and meta-analyses' – 2019

The lengthy 2019 review concluded that the 11 RCTs of ECT for depression (remember, there have only been 11) were so flawed that the five meta-analyses were wrong to draw any conclusions about whether ECT is or is not effective (Read et al., 2019*a*). Like NICE (2003) it called for better research. Unlike NICE, it also called for a suspension of ECT until such research is forthcoming.

### Narrative or systematic?

Meechan *et al*.'s main attack strategy, again, is to seek to discredit the entire paper by claiming it is not a systematic review; but they then go on to evaluate it as if it was.

Meechan *et al*. assert that the quality of our review would be scored ‘critically low’ by a particular evaluation method for *systematic* reviews called AMSTAR-2 (Shea *et al*., 2017). Their supplementary material shows that they scored our review as having failed on four of AMSTAR-2's ‘critical domains’. The only one they mention in the body of the manuscript, so perhaps their biggest concern, is that we failed to pre-register a protocol for our review. This is true, and fair criticism. Neither the Semkovska and McLoughlin (2010) review discussed earlier, nor the recent meta-analysis (Mutz *et al*., 2019), both of which involved co-authors of the Meechan *et al*. paper (Mcloughlin and Young respectively), had pre-registered protocols. Meechan *et al*. failed, as far as I am aware, to pre-register a protocol for their own meta-analysis. In their defence, however, and ours, a recent review found that ‘About half of the surveyed systematic reviews’ authors have never registered any of their SRs' protocols' (Tawfik *et al*., 2020). Furthermore, registration, while desirable, does not, as implied, equate to high quality. There are high and low-quality reviews in both groups.

The second and third of the four domains we supposedly failed are related to each other: ‘Did the review authors use a comprehensive literature search strategy?’ and did they ‘provide a list of excluded studies and justify the exclusions?’ (Shea *et al*., 2017). We actually reported two systematic reviews in the 2019 paper, one of the RCTs and one of the meta-analyses thereof. For some reason Meechan *et al*. do not apply AMSTAR criteria to the latter. In relation to our review of the meta-analyses (pp. 65, 68, 80, 85–90, 92), we stated that:

A medline (MESH) search for meta-analyses on the effectiveness of ECT for depression using placebo-controlled trials (ECT *v*. SECT), was conducted in June 2019, using the following index terms: [‘ECT’] OR ‘electroshock therapy’ OR ‘electroconvulsive treatment’ OR ‘electroshock treatment’ AND [‘meta-analysis’] AND ‘depression’ or ‘major depressive disorder’] (Read *et al*., 2019*a*, p. 65)

We also provided a flowchart of the search strategy (p. 68), which included a list of the nine papers excluded from the 14 that had been deemed potentially ‘eligible’.

Our other review in the 2019 paper, of the RCTs, clearly states that the focus is on ‘ECT versus sham ECT trials’ (p. 64), and that the studies to be included were those ‘cited by the meta-analyses’ (p. 65). As established earlier there have only ever been 11 ECT *v*. sham ECT RCTs. None of the five meta-analyses we reviewed, including the most recent (Mutz *et al*., 2019), identified any other RCTs beyond these eleven. An informal search by the first author, just before submission of the 2019 paper, confirmed that the meta-analyses, and his own previous review (Read and Bentall, 2010), had not missed any studies Nevertheless, we should have conducted the search formally, again, and reported the process in our latest review.

The fourth ‘critical domain’ on which we are judged to have failed was ‘A satisfactory technique for assessing the risk of bias in individual studies that were included in the review’ (Shea *et al*., 2017). In fact, the bulk of the review was devoted to assessing the quality and bias of the eleven RCTs (and the failure of the meta-analyses to take those into account). We developed a 24-point quality scale to do so, discussed next. Meechan *et al*. have now conducted their own assessment of the studies, using the Cochrane Risk of Bias 2 tool (Sterne *et al*., 2020). Although I might quibble over some of their scoring, this is a very valuable contribution to the literature, and I thank them for their work on this. It certainly deserves better than to be buried in a supplementary file. When two quite different methods reach broadly the same conclusion, about the very poor quality of the studies in question, one can be reasonably confident about that conclusion.

Another accurate criticism, on a non-critical AMSTAR domain, was our failure to have more than one of us conduct the literature reviews. Fortunately, since everyone in the field agrees how many RCTs and how many meta-analyses exist, this failing on our part did not result in anything being missed or wrongly included.

### The quality scale

Meechan *et al*. pay much attention to our use of an ‘unvalidated quality scale’. We were unable to use a previously validated scale, which would have been desirable, because, in keeping with the pro-ECT community's disinterest in the quality of ECT efficacy studies, no such ECT-specific scale has ever been developed in the 70 years that ECT has been researched.

Our scale was designed, in the absence of any pre-existing instrument, specifically to assess RCTs of ECT for depression. Each of the 24 Quality Criteria was carefully defined and the definitions reported for readers to assess, in a table (p. 66). The 24 items included traditional, general criteria for RCTs (randomisation, blinding, incomplete outcome data and selective reporting) plus some ECT specific items.

The 11 studies were independently scored, blind, by two raters. Rather than acknowledge that this is desirable, and does not always occur, Meechan found a scale that places our level of inter-rater agreement in the ‘weak’ category (by one percentage point) rather than in the middle of the ‘fair to good’ range according to the scale reported by ourselves. Meechan *et al*. fail to mention that the scores of the two blind raters for the 11 studies were highly correlated (*p* = 0.001). Nor do they mention that the discussion between the raters to resolve the discrepancies led to increased Quality scores.

Read *et al*. are criticised for not defining a cut-off score ‘demonstrating what would constitute a good or poor quality trial’. One wonders how Meechan *et al*. would have reacted if we had done so. Presumably, we would have been accused of ‘unvalidated’, ‘arbitrary’, ‘subjective’, ‘biased’ decision making. We were very clear that all the studies were of poor quality, which was plain to see by any neutral observer, and described them only in relation to one another, with terms like ‘lowest quality’.

### Scale items and scoring

Our Quality Scale is criticised for having only a binary ‘yes’ and ‘no’ scoring system instead of including an ‘unclear’ option. This is a fair point, in relation to some of the items. Meechan *et al*. wrote:

To take a specific example, for the item ‘Decliners described’ (i.e. any description of people who were approached, but declined to participate), multiple studies are negatively scored because they do not report on any decliners (e.g. Lambourn and Gill, 1978, West, 1981). Under Cochrane guidance, the appropriate response is ‘unclear’.

This item, like many others, has no possible ‘unclear’ option. Either the study had a description of people who declined to take part in a study when invited, or it did not. The issue of decliners is an important methodological issue. If, say, only 50% of the ECT recipients who were asked to take part in an ECT study agree to do so, then the participants cannot be said to be representative of ECT patients. Rather than acknowledge that it is problematic that only three of the eleven studies meet this basic methodological standard, Meechan *et al*. choose instead to quibble, illogically, about the scoring of the item.

Meechan *et al*. continue:

The scoring and scaling introduces further biases against accurate reporting of quality. For example, pooling of age and gender into a single criterion is baffling – these two features are independent. The scaling used means failing on either one alone is a failing on both and therefore biases against fair quality assessment.

This item assessed whether the studies' samples represented the demographics of people who actually receive ECT today and, therefore, whether their findings are meaningful. In England, for example, twice as many women as men are given ECT, and most ECT recipients are over 60 years old (Read et al., 2018; Read *et al*., 2021*b*). The definition of the criterion (which was explicitly stated) was rather generous: ‘More than 50% female (but not all), and mean age of 50 or more.’ Meechan *et al*.'s argument is not unreasonable. Yet it would result in awarding a quality point for representativeness to the study that met the age criteria but was 100% male, as well as to the three studies that met the gender criteria but actively excluded older people. Only the three studies that met both the gender and age requirements would have received an extra quality point if Meechan *et al*.'s concern was acted on, while the denominator for all 11 studies would be increased from 24 to 25. So, the average quality performance would be no different (12.54/25; 0.50) than the one we reported (12.27/24; 0.51). It is a shame, again, that Meechan *et al*.'s concern about the scoring of this item was not matched by just a modicum of concern that only three of the eleven studies used to support the claim that ECT is effective were conducted on the sort of people on whom it is actually used. (Responsible researchers and clinicians might also show some concern that older people and women are disproportionately exposed to this treatment, despite their being at higher risk of memory loss (Sackeim *et al*., 2007)).

Readers are told that ‘The inclusion of some items seems unjustified and runs the risk of biasing the scale in favour of the authors’ possible preconceived beliefs about the trials'. Unfortunately, Meechan *et al*. provide just two examples of which of the 24 items meet their undefined criterion of ‘unjustified’ and should therefore be left out of evaluations of ECT studies. These two items, quality of life scales and validated depression scales, are deemed ‘unjustified’ because the studies ‘were conducted prior to the development of any of these scales’. This bizarre line of argument implies that the findings of very old research studies conducted before methodological standards were improved should not be judged by the new standards and should, when making decisions about patient safety, be given as much weight as those conducted using those new standards.

It should be noted, given Meechan *et al*.'s objection to the inclusion of a quality of life measure in the 24-point study Quality Scale, that in 2003 NICE announced:

More research is also needed to determine the cost-effectiveness of ECT. In particular, better quality-of-life information is needed for people considered for, or who have received, ECT.

No RCT for ECT has ever measured the quality of life of ECT patients, in the 65 years before the NICE recommendation or the 19 years since. Meechan *et al*. could have demonstrated that they are genuinely concerned about quality research and patients' wellbeing by echoing the NICE recommendation and urgently calling for such studies. Instead, they bemoan the inclusion of the issue as a quality criterion and try to convince readers that doing so was ‘biasing the scale in favour of the authors’ possible preconceived beliefs about the trials.

I also do not agree that items such as ‘patient ratings’ and ‘suicide measures’ are necessarily less important than other items.

Later, Meechan *et al*. try to argue that the studies ‘would likely not be consistent with contemporary standards yet are still of a high enough standard to justify conclusions drawn.’ They offer no evidence or logic for such a curious claim. Despite all the evidence, provided in great detail by the two reviews, that the studies come nowhere near being of a ‘high enough standard’ Meechan *et al*. feel they can just assert the opposite and that doing so somehow makes it true.

### Misunderstanding?

Meechan *et al*.'s next section is titled ‘Misunderstanding the nature of Evidence-based Medicine (EBM), placebo and active comparators’. This allegation suggests to the reader that the authors of the review might be either ignorant of scientific processes and placebo effects, or unintelligent, or both. Meechan *et al*. support this rather silly assertion about our inability to understand things, by claiming that:

…it represents misunderstanding of principles of EBM that both Read and Bentall (2010) and Read *et al*. (2019*a*) focus exclusively on ECT-sECT RCTs and their associated meta-analyses as the only relevant evidence. The authors fail to consider these studies within the wider context of other relevant evidence

This is untrue. The first paragraph of Read *et al*. (2019*a*, p. 65) states quite clearly that:

‘….ECT must be assessed using the same standards applied to psychiatric medications and other medical interventions, with placebo-controlled studies as the *primary* method for assessment. {italics added}

Furthermore, the first paragraph of the Read *et al*. (2019*a*, p. 65) review also refers to ‘The many recent studies that either compare ECT to other treatments, or compare different types of ECT with each other (Read and Arnold, 2017)’. Thus, it is blatantly obvious that we were not suggesting that RCTs are the only type of evidence, or that there is no other evidence to consider. But the focus of both reviews was, indeed, the ten or 11 RCTS, precisely because it is well established that such studies are extremely important.

### Are RCTs necessary?

Faced with the absence of the robust RCT evidence that is, today, required for all medical treatments (Read and Moncrieff, 2022) most branches of medicine would try to fill the embarrassing void, as quickly as possible, with a series of robust studies. But ECT advocates, instead, repeatedly tell us that the lack of robust RCT evidence really doesn't matter, because there are lots of other studies that will suffice, such as comparisons between different types of ECT or between ECT and other interventions. This is unacceptable.

Moreover, the review of the non-placebo studies that we cited (Read and Arnold) had found that none ‘produced robust evidence that ECT is effective for depression, primarily because at least 60% maintained ECT participants on medication, 89% produced no meaningful follow-up data beyond the end of treatment, and none investigated whether ECT prevents suicide.’ (Read and Arnold, 2017). Meechan *et al*. don't mention that review.

Let us examine a very recent example of the kind of non-placebo study that Meechan *et al*., and other ECT supporters, rely on as evidence that ECT is effective, and safe. Swedish researchers (Ekstrand *et al*., 2021) assessed the efficacy and safety of ECT on 91 depressed ECT recipients (compared to ketamine). The absence of a placebo group was justified, as usual, by arguing that it would have been ‘not ethical’ to withhold ECT. This frequently proposed argument, that one can't research whether × works because one just knows that × works and therefore × cannot be withheld from distressed people in order to find out whether × works, positions ECT proponents beyond the parameters of science and evidence-based medicine (negated by Read *et al.* (2021*a*); Read and Moncrieff, 2022; and see Table 1).

**Table 1.** The six defences against the continuing absence of any evidence of efficacy from adequate randomised, placebo-controlled studies (RCTs) of ECT

1	ECT has been used for a long time so we know it works
2	It's unfair to critique the pre-1986 RCTs using today's scientific standards
3	It's unethical to conduct RCTs that involve withholding treatment from very ill people
4	RCTs aren't necessary; non-placebo studies are sufficient
5	ECT *is* effective long-term, if you use antidepressants after ECT ends
6	Denigrate the people raising the issue, or scientific/media outlets publishing their critiques

In the Swedish study, as in most other non-placebo ECT studies (Read and Arnold, 2017), most participants (94%) continued to use psychiatric drugs (85% were on antidepressants), thereby rendering any conclusions about the reasons for any improvement unconvincing. All assessments, of efficacy and adverse events, were not only not blind to which group the participants were in, but were actually conducted by the psychiatrists administering the treatments. This study was, however, still better than most of its kind (Read and Arnold, 2017) because it did provide some follow-up data. Even under the extremely subjective and potentially biased assessment process, more than one in three ECT patients (37%) were assessed as having not improved at the end of the course of 12 ECTs. Of those who did improve, 50% relapsed within 6 months and 64% within a year. So, only about one in five people (22%; 20/91) gained any lasting benefit from the treatment. Meanwhile, six of the 57 who did improve during treatment (10%) tried to kill themselves within a year and those suicide attempts were rated as ‘very likely’ or ‘probably’ related to the treatment. One died from the attempt, 3 months after ECT. The researchers did not report how many of those who did not improve during treatment tried to kill themselves. Presumably, this was even higher than 10%. Ketamine was even worse than ECT in the short term, but equally poor in terms of relapse or suicide attempts. Most studies (placebo and non-placebo alike) have no follow up data, thereby ignoring ECT's massive relapse rates (Tew *et al*., 2007; Nordenskjold *et al*., 2011). But even when follow up is conducted, and the relapse and suicide rates are thereby exposed, the researchers conclude, like so many authors of non-placebo studies before them, and like Meechan *et al*., that because a comparison treatment performed worse than ECT during the treatment period, ECT ‘remains the most effective treatment for severe depressions’ (Ekstrand *et al*., 2021).

### The six defences

This pretence that the absence of evidence from RCTs is unimportant because we can rely on non-placebo studies is one of the six standard defences used by ECT advocates against any suggestion that the absence of |robust evidence is a major problem (Table 1); (Read and Moncrieff, 2022). Meechan *et al*. also deploy defence number 2: ‘It's unfair to critique the pre-1986 RCTs using today's scientific standards’ (see above), and defence number 1 ‘ECT has been used for a long time so we know it works’. Meechan *et al*.'s very first sentence is ‘Electroconvulsive therapy (ECT) has been used in the treatment of major mental illness, particularly depression, since its initial recorded use in 1938’.

Trying to persuade readers that Read *et al*. do not understand the nature of Evidence-based Medicine (EBM) and placebo effects, would seem to be an example of defence number 6: ‘Denigrate the people raising the issue’.

### Blinding and the placebo effect

Finally, Meechan *et al*. take exception to our pointing out that the inclusion of people who had previously received ECT renders studies unblinded ‘because they know that ECT is always followed by headaches and disorientation’ (Read *et al*., 2019*a*, p. 89). We concluded that ‘by not excluding people who have previously had ECT all 11 studies exaggerated the difference between ECT and SECT in ECT's favour, and that none were truly blind studies’ (p. 97). Meechan *et al*. challenge the fact that ECT has very common distinguishing effects immediately after regaining consciousness. The only reference they offer is a report (Scottish ECT Accreditation Network, 2020) which relied on the treating psychiatrists' subjective assessments to conclude that the incidence of post-ECT headache is only about 30%. Research studies, and anyone, like myself, who has sat with many ECT recipients as they come round (Read, 2021*d*), would suggest otherwise. For example, the recent Swedish study mentioned earlier reports that ECT patients experience an average of eight adverse effects, with 94% suffering at least one. Muscle pain was suffered by 53%, and headaches were experienced by 80%.

We had pointed out that five of the studies definitely included people who had previously had ECT and noted that none of the other six studies had stated whether they did or not. It seems likely, however, that if they had gone out of their way to exclude such patients, they would have said so. But for some reason, Meechan *et al*. conducted an analysis comparing the two groups as if there was a definite difference between them on this matter, which is unlikely. They also failed to report our unique and careful analysis of the only study ever to have identified two groups that definitely had, and had not, previously received ECT (Lambourn and Gill, 1978), which supported our hypothesis. More studies, with larger samples, are necessary on this crucial topic.

## Conclusions

The serious problems with the evidence-base for ECT have been elaborated elsewhere (Ross, 2006; Rasmussen, 2009; Read *et al*., 2013, 2019*b*; Moore, 2020; Read, 2021*d*; Read and Moncrieff, 2022), as have flaws in its administration in England (Harrop *et al*., 2021; Read *et al*., 2018, 2021*b*). The personal stories of ECT recipients, however, are rarely gathered and reported (Wells *et al*., 2021).

In order to remind us that this is not just an academic debate about methodology and ideology, I offer just one such personal story, to represent the stories of the thousands of people around the world who have been damaged, temporarily or permanently, by a treatment that provides no hope of any long-term benefit. There are, of course, stories from people who believe ECT saved their life, and all concerned are pleased for them. I believe, however, that psychiatry has an ethical responsibility to listen less defensively to the thousands who are still waiting for an acknowledgment of the damage done, let alone an apology, compensation or rehabilitation. Amidst a barrage of vitriolic comments (Vlessides, 2020) about the authors of the 2019 review, our profession, the journal that published our review and even the media platform that reported on it, *Medscape,* Dr Sandra Brink wrote:

Today I resent myself for agreeing to receive ECT. My long-term memory was destroyed. Memories of childhood friends, memories of major events I attended, memories of my training as a psychiatric registrar, academic memories etc. I started struggling with simple spelling and calculations. I basically cannot recall an almost entire 3 years (2004–2006), including the relationship I was in at the time. I never told colleagues about this, as I felt ashamed. But I started talking to other people who had ECT and realized I am not alone. ECT robbed me of precious memories. And the inability to recall often caused embarrassment. In the 20 years after qualifying I have never treated a patient of mine with ECT. It will have to be a life vs death situation before I even consider it. The facts are real. You have permanent amnesia, retrograde and anterograde. I can understand some of the negative response by colleagues to this article, but I have to admit that I welcome their argument. (Brink, 2020)

I conclude by repeating the findings of our 2019 review, lest we lose sight of what I believe are important truths amid the repetitive and tiresome, but necessary, defence of our reviews:

The quality of most SECT–ECT studies is so poor that the meta-analyses were wrong to conclude anything about efficacy, either during or beyond the treatment period. There is no evidence that ECT is effective for its target demographic – older women, or its target diagnostic group – severely depressed people, or for suicidal people, people who have unsuccessfully tried other treatments first, involuntary patients, or children and adolescents. Given the high risk of permanent memory loss and the small mortality risk, this longstanding failure to determine whether or not ECT works means that its use should be immediately suspended until a series of well designed, randomized, placebo controlled studies have investigated whether there really are any significant benefits against which the proven significant risks can be weighed. (Read *et al*., 2019*a*, p. 64)

We just don't know whether ECT is better than, worse than, or no different from, placebo (p. 101).
